# Perception of Gender-Based Violence and Sexual Harassment in University Students: Analysis of the Information Sources and Risk within a Relationship

**DOI:** 10.3390/ijerph17113754

**Published:** 2020-05-26

**Authors:** Mercedes Osuna-Rodríguez, Luis Manuel Rodríguez-Osuna, Irene Dios, María Isabel Amor

**Affiliations:** 1 Department of English and German Philology, University of Cordoba, 14071 Cordoba, Spain; mercedes.osuna@uco.es; 2 Department of Education, University of Cordoba, 14071 Cordoba, Spain; d52roosl@uco.es (L.M.R.-O.); m.amor@uco.es (M.I.A.); 3 Department of Psychology, University of Cordoba, 14071 Cordoba, Spain

**Keywords:** student training, gender studies, gender-based violence, sexual harassment, university

## Abstract

In a truly democratic society, there should be no place for any kind of discrimination or violence. Among the basic tools for eradicating discrimination and violence against women, education has a crucial role to play. Education about gender should be considered at all levels, in all year groups and across the curriculum, so as to improve education about this subject. Although these matters are increasingly addressed, at university level, including at postgraduate level, they are often forgotten. The purpose of this study is to break down the level of knowledge of gender-based violence and/or sexual stalking, the sources of information most widely used for developing this knowledge, and beliefs about situations of risk in relationships among a sample of 268 Science and Social Science students at the University of Córdoba (Spain). The analysis was descriptive, comparative and correlative. Means and standard deviations were analyzed, and correlations were used to establish possible relations among the variables. Cluster analysis was used to distribute the sample with respect to knowledge of violence and Student’s *t*-test was used to identify differences between groups. The chi-squared test was used to find the association between variables such as situations of violence and places of residence. The results show that, although the experience of gender-based violence is among the least common sources of violence, there is evidence that these situations do exist, and the risk of violent acts and/or stalking is greater when couples break up. The perception of risk is higher when students have a greater knowledge of gender-based violence or sexual harassment and this perception is higher in women. As expected, greater knowledge is also associated with experience of this type of situation; however, place of residence was not linked to greater or lesser knowledge. Training in gender is considered essential and necessary in the university environment.

## 1. Introduction

It is clear that, despite progress recognized by legislation, inequalities between men and women still exist in the 21st Century [1]. The patriarchal figure of the “white European male” still exists against all logic and justice and despite all social and legal progress.

While it is true that simply being a woman leads to discrimination, it is also true that a variety of factors may contribute to double or even multiple discriminations [2], for example in the rural versus the urban environment, in functional diversity, ethnicity, religion, skin color, nationality, sexual identity, etc. [3,4]. These discriminations show up in varying degrees and in different areas, the cruelest result being murder.

As Kate Millett suggests when she says that the personal is political [5], discrimination against women in whatever form, and the violence practiced against them, is a political question that involves the whole of society and requires social, political and legal measures. According to Korac [6], the construct of men as “protectors”, and being in that sense “heroic”, has been one of the central pillars of the oppressive gender systems inherent in the patriarchal structures of states, as well as in the socioeconomic and political systems that consolidate them.

Undoubtedly, if a society truly wishes to be democratic, it must respect human rights [7], and there is, therefore, no place for any type of discrimination or aggression against women merely for being women.

The measures and strategies used to eliminate discrimination and gender-based violence against women must be based on two main axes: the political and the educational one. In the political sphere, it is essential that legislation and legal regulations aimed at preventing and combating gender-based violence are clearly defined to avoid possible inaccuracies that may expose important dimensions and victims within these situations. According to the ViDaCS (Violent Dads in Child Shoes) project, there is a need for an integrated and holistic theoretical and operational model in order to understand gender-based violence as gender-based violence and this intervenes with the objective of ending the fragmentation of existing measures. This ecological model assumes that individual wellbeing can only be achieved if relational, organizational and collective levels are integrated and it proposes functional connections between different services and specific preventive initiatives [8].

In the educational environment, it should be introduced at all levels in all year groups and across the curriculum [9], so as to reduce the educational deficiencies concerning gender at the family level. In this sense, recently, several authors detect among the results of their research that gender-based violence is a phenomenon that occurs recurrently among university students [10]. Among the different typologies stands out bullying, violence and physical-sexual harassment as forms of violence; those responsible are different male members of the education community such as teachers and/or peers.

This implies a carefully drawn-up plan at all levels of education. Nevertheless, when speaking of levels or stages of education, the university level, including the post-graduate level, is often forgotten [11]. In this sense it should be remembered that there have been so many centuries of discrimination and abuse [12] that it is not enough to educate in equality at just some educational levels and between a few widely spread activities, but rather that this training should be part of education at all age levels and should be cross-curricular and present in all areas and subjects in order to mitigate the patriarchal education that has been received for so long. In fact, some research has shown that intervention is necessary at the university level and, specifically, in initial courses of university education [10]. In this regard, it is essential to promote the correct self-esteem and self-confidence among university women as a brake on possible sexual coercion. In this sense, training in risk recognition of situations of violence is absolutely important in order to reduce the likelihood of gender-based violence. A recent study [13] undertaken in universities in Arequipa (Peru) shows benevolent sexism among male students, and that female students are more aware of the risks of gender-based violence and sexism in universities.

Our main purpose is to transfer this to other contexts and to reduce this type of situation in any field.

In the study conducted by Igareda and Bodelón [2] in Spanish universities relating to students’ experiences in facing sexual violence they attempt to understand why these experiences are not communicated or reported. Women recognize that university life constitutes a new context in which specific forms of sexual violence are developed. University life includes profound changes for young women: living outside their parents’ house, being free to enter and leave, having “atypical” schedules, etc. These changes imply transformations in the way these women live, allowing some of them to break gender stereotypes expected by society [14]. However, the new context of autonomy that university life entails does not always make it easier to identify situations of violence against women. The difficulty of recognizing themselves in situations of gender violence is frequent. To the difficulty of identifying the phenomenon, topical issues are added such as the fact that these situations do not occur among university women, or that their status as university students should have enabled them to realize earlier about what was happening. All this can contribute to generating feelings of guilt. The difficulties students have in identifying what constitutes sexual violence has also been confirmed in similar research within Spanish universities [1,12], as well as in research on sexual violence in general [15]. However, in Italy the università in rete contro la violenza di genere {UN.I.RE Project (2018–2020)} to prevent and combat violence against women and domestic violence aims to improve the implementation of the Council of Europe Convention [16] through a recognition and consolidation of the roles that universities and the academic world can and should assume. This project is led by the University of Milano-Bicocca and it develops research to help develop prevention and identification strategies concerning gender-based violence and training aimed at students to promote training and strengthening skills to combat gender violence.

It is thus of vital importance to know the constructs of gender [17] within the student body, specifically concerning violence against women [18]. The ideas underlying the collective imagery of future citizens also includes nuanced and important variables such as whether the sources of knowledge about gender-based violence are the same in rural areas as they are in the urban environment [19]. This is a matter of great importance as often the media do not respect the agreed code of practice when presenting news stories, allowing their sexist beliefs to come through which in turn strengthens patriarchal ideas.

It is also important to produce a detailed analysis of what university students know about gender-based violence [9], and of how many people close to them they know to have suffered it. Deconstructing societal beliefs and patriarchal myths is the only way of eradicating behaviors which may ultimately result in death [5]. This detailed breakdown will also be the standard for designing strategies in order to implement plans to be carried out in all areas and at all educational levels [20] in order to achieve effective equality between men and women [21]. Only through knowledge and training can the idea spread through society that women and men should enjoy the same human rights with the same opportunities.

In essence, this comes down to women being able to occupy the public space without society feeling that they are invading a male space, and equally that men can occupy the private space that has, up to now, always been considered an area that is almost exclusive to women.

The union of these two areas—the public and the private—will erase socially constructed differences, and with them the greatest expression of these inequalities: gender-based violence [3,4,7].

The use of education to prevent gender-based violence against women is of increasing importance in our society and has become more of a need than a right for women, and has become a social duty [22] due to the events that occur day after day. In September 1995 the Beijing Platform for Action passed, in the Fourth World Conference of Women of the United Nations (UN), a definition of gender-based violence as being any violent act against those belonging to the female sex which results or might result in physical, sexual or psychological damage to the woman, which includes threats, coercion or the deprivation of liberty. It further clarifies that violence against women is of itself a violation of their human rights and an attack on their fundamental freedoms that contributes to social, political, economic, and cultural inequality between men and women, and its continued existence has been permitted by legal and political systems that have discriminated against women throughout history.

In 1993, the General Assembly of the UN passed an historic resolution, specifically the “Declaration on the Elimination of Violence against Women” [23], which has led to a series of resolutions related to matters dealt with in the Human Rights Commission [24,25]. All these texts recognize the relation between gender-based violence and the social situation of discrimination against women—that is, gender inferiority [26]—which has been statistically confirmed. The aim of the international standards is to require the member states of these organizations to take appropriate and effective preventive and punitive measures to eliminate all forms of violence in any area in which it may occur. Violence against women may take place in private or in public, in domestic, social or working environments [27]. Likewise, there are national and international documents which demand equality between men and women and mutual respect at all stages of the process of socialization and shared living, from the time that education begins. Education systems should ensure that this is a reality [28] and not just a decision. This opinion is shared by all citizens, as shown by studies and research carried out so far [9,29].

Within the research performed in Spain into gender-based violence in the university environment [30,31], there are studies not limited exclusively to sexual violence, but which address gender-based violence in general, or study some particular form of gender-based violence such as sexual stalking and stalking for sexual purposes. Most of these studies into sexual violence in universities study the Anglosphere, and to a lesser extent, other European countries [32,33,34,35]. They mostly analyze different ways in which gender-based violence may be expressed in universities [1] and examine abusive and discriminatory behaviors towards women in the university environment. In this university context and after the review of several pieces of research, we found a common problem independent of the field of study from research focused on degrees related to the Social Sciences, such as those related to Health Sciences [36]. Specific focus should be given to studies into teacher training [37,38,39,40], highlighting the need to incorporate education into gender and sexual diversity in initial teacher training, as they will have a primary responsibility for developing a comprehensive education in their future students [38].

Many researches point out the intervention and training of students as tools for the prevention of risk situations [41]. Given this reality, many universities are implementing measures to avoid situations of gender-based violence and to raise awareness of sexist beliefs or negative relationships among university students. These measures include support and counseling programs in the different university degrees [1].

On the basis of the above, having viewed the scientific literature and the research carried out on gender-based violence and sexual harassment, this study intended to go more deeply into the thoughts and values of the student body with regard to these matters in the university environment. The data are assessed to analyze the level of knowledge about gender-based violence and/or sexual harassment, the sources of information most widely used in acquiring this knowledge, and the beliefs about possible situations of greater or lesser risk within a relationship.

## 2. Objectives

1. Analyzing the relationships between knowledge about violence situations, sources of information and risk situations in couple relationships among university students.
2. Creating profiles among students about the knowledge of violent situations to detect differences between the sources of information used and their perception of risky situations.
3. Checking the relation between the knowledge of risky situations of gender-based violence and the place of residence (rural vs. urban) and the sex of the participants.

## 3. Methodology

### 3.1. Participants

A total of 268 students on Bachelors or Masters Degree courses at the University of Córdoba (Spain), in the fields of Education (21.6%), Biology (30.6%), Biochemistry (5.2%), Geography and History (12.7%), Biomedicine (5.2%), and Veterinary Science (24.6%) took part in the study. The sample obtained was incidental; those students who were in the classroom at the time of the surveys participated. Of this total, 66.4% (*n* = 178) were women and 33.6% (*n* = 90) men, and their ages ranged from 18 to 30 (M = 20.39; SD = 1.780). With regard to the family home of these students, 70.5% came from an urban environment and 29.5% from a rural.

### 3.2. Instrument

To gather the information, an ad hoc questionnaire was constructed, which retrieved the sociodemographic data of the participants, and asjed questions related to the most widely used sources of information where knowledge about gender-based violence and sexual harassment can be gained (e.g.,: “TV News/Television”).

The instrument used was called “Situations of Risk in a Relationship” (S-SRR) related to situations of greater risk for acts of gender-based violence or sexual harassment at different points of a relationship (e.g.,: “As boyfriend/girlfriend”). This instrument was presented via a single-factor Likert-type scale from 1 (“Never”) to (“Always”), comprising six items.

A second instrument was then used, called “Knowledge of Situations of Gender-based Violence” (S-KSGV), to measure whether someone knows of anyone around them who is experiencing some kind of act related to gender-based violence or sexual harassment (e.g., “Threatening or Intimidatory Messages or Emails”). This instrument was presented via a single-factor Likert-type scale from 1 (“Never”) to (“Always”), comprising twelve items.

Both instruments (see Table 1) show good internal consistency, as they have Cronbach’s alpha values between 0.70 and 0.90, which is understood as acceptable in unidimensional scales [42].

### 3.3. Procedure and Analysis

The questionnaires were filled out during class time, and answers were individual and voluntary, taking about 15 min. The participants in the study were informed of the confidential treatment of the data provided, the anonymity of the study, and the voluntary nature of participation. All the students were above the age of majority. No incidence was registered in the completion of the survey and no participant refused to answer the questions.

### 3.4. Analysis of Data

Descriptive and comparative statistical analyses were performed using version 20.0 of the SPSS software. Descriptive analyses were carried out first, examining the mean values and the standard deviations of the variables. Next, bivariate correlations were used to establish possible relations between the different variables under study: (a) Knowledge of situations of gender-based violence and sexual harassment; (b) Situations of risk in a relationship; and (c) Sources of information used to acquire knowledge.

Subsequently, a cluster analysis was performed to distribute the sample into groups with respect to the valuations given by the participants concerning knowledge of situations of violence on the part of partners or ex-partners. Once the sample was distributed, Student’s *t*-test for independent samples was used to detect possible differences between groups arising from the cluster analysis, and their valuations of information sources most widely used to obtain knowledge on gender-based violence or sexual harassment, and possible situations in a relationship where there is greater risk of these acts taking place.

Likewise, a chi-squared test was used to confirm the association of these variables within the knowledge of situations of gender-based violence and the place of residence of participants. Cramer’s V was used to determine the strength of the association, bearing in mind the typified corrected residues in the contingency tables for determining the relations between variables.

Finally, Student’s *t*-test was used to analyze differences in the perception of violent situations, sources of information, and situations of risk on the basis of gender. The effect size of the results obtained from Student’s *t*-test (Cohen’s d) was evaluated, with values <0.20 considered small, >0.20 and <0.50 moderate, and >0.80 large [43]. The significance level is 95% (*p* < 0.05) and 99% (*p* < 0.01), depending on the case.

## 4. Results

The results obtained according to the proposed objectives are shown as follows:

### 4.1. Descriptive Results on Knowledge of Situations of Violence, Sources of Information, and Situations of Risk in Relationships

**Figure 1:**
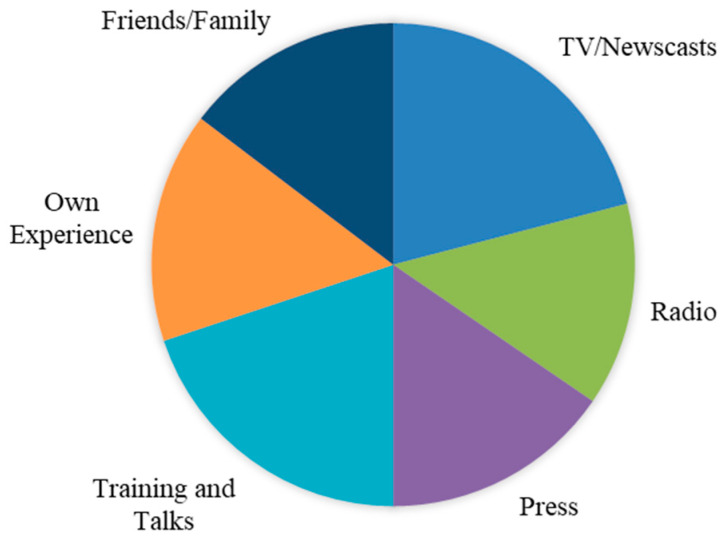
Knowledge of situations of violence as a function of sources of information. TV = TV news/Television.

In general, students valued sources of information related to the news (TV newscasts, etc.) (M = 3.43; SD = 1.038), with training and talks (M = 3.26; SD = 1.193) and the press (M = 3.53; SD = 1.136) being the means most used to acquire knowledge on gender-based violence (see Figure 1), as opposed to less-used sources such as their own experience (M = 2.53; SD = 1.428) or the experience of friends and/or family members (M = 2.40; SD = 1.417).

**Figure 2:**
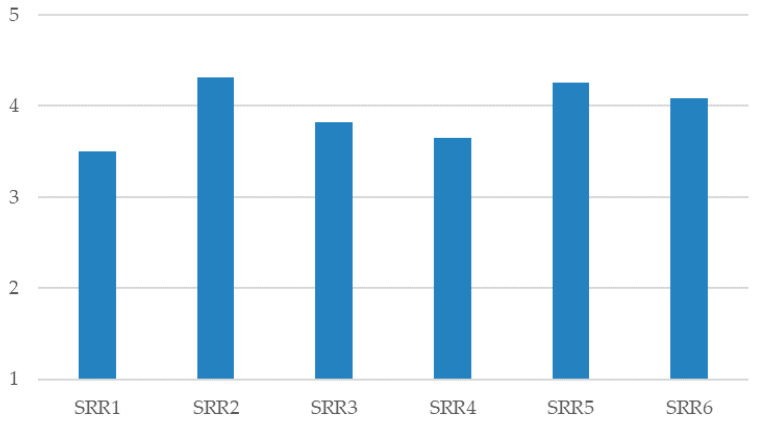
Values for situations in a relationship with risk of acts of violence. SRR = “Risk Situations in a Relationship”.

They considered that situations of greater risk of acts of gender-based violence or sexual harassment (Figure 2) may occur during the divorce/separation of a couple (M = 4.08; SD = 0.980) or afterwards (M = 4.01; SD = 1.049) and in trying to break up a boyfriend/girlfriend relationship (M = 4.34; SD = 0.866).

**Figure 3:**
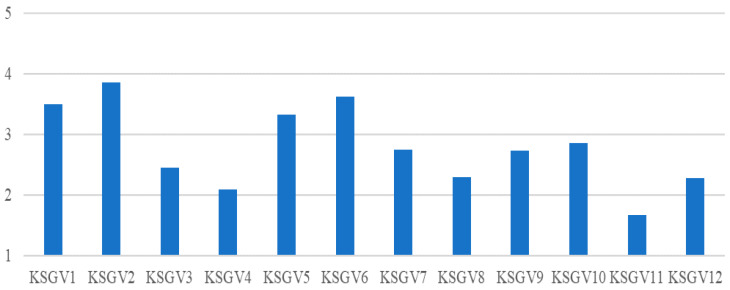
Type of knowledge about situations of gender-based violence. KSGV = “Knowledge of Situations of Gender-based Violence”.

With respect to the knowledge of situations of gender-based violence suffered by people in respondents’ circles (see Figure 3), participants expressed greater knowledge of situations of jealousy or possessive feelings (KSGV2: M = 3.85; SD = 1.194), followed by sexist remarks (KSGV6: M = 3.62; SD = 1.328), constant control of activities or relations with other people (KSGV1: M = 3.49; SD = 1.367), psychological attacks (KSGV5: M = 3.33; SD = 1.450), obscene remarks, rumors or attacks on their sex life (KSGV10: M = 2.85; SD = 1.469), and pressure to engage in an emotional and/or sexual relationship (KSGV7: M = 2.75; SD = 1.398).

On the other hand, those situations about which they have the least knowledge are those related to preferential treatment or academic favors in exchange for sexual favors (KSGV11: M = 1.79; SD = 1.348), followed by physical attack (KSGV4: M = 2.29; SD = 1.510), demeaning use of Internet images, even as a “joke” (KSGV12: M = 2.27; SD = 1.412), kissing and/or touching without consent (KSGV8: M = 2.14; SD = 1.330), threatening or intimidatory messages or emails (KSGV3: M = 2.36; SD = 1.483), and discomfort or fear as a result of feeling harassed or intimidated (KSGV9: M = 2.73; SD = 1.479).

### 4.2. Correlations between Variables Referring to Knowledge about Situations of Violence, Sources of Information, and Situations of Risk in a Relationship

The results of the bivariate correlation analyses show significant relations between some of the variables about knowledge of situations of violence, sources of information, and situations of risk in a relationship (see Table 2). Specifically, a positive correlation was found between the scale for the knowledge of situations of violence, and the situations of risk in a relationship (r = 0.326; *p* < 0.01).

Although no correlation was found between the situations of risk in a relationship and the most common sources of information (such as television, radio, etc.) for obtaining knowledge on gender-based violence and sexual harassment—except a positive relation with experience of family members and/or friends (r = 0.232; *p* < 0.01)—positive correlations were found between these sources of information and the knowledge of situations of violence. Specifically, knowledge of situations of violence was positively related to sources of information based on experience of the respondent (r = 0.281; *p* <0.01) and/or some family members (r = 0.460; *p* < 0.01), the press (r = 0.171; *p* <0.01), and training and talks received (r = 0.150; *p* < 0.01).

### 4.3. Profile of Student Knowledge on Situations of Violence and Differences as a Function of Sources of Information and Situations of Risk

Subsequently, the cluster analysis divided the sample into two groups, one characterized by having a profile of greater knowledge about situations of violence experienced by people close to them from a partner or ex-partner (*n* = 169; 63.5%), and another by having a profile with a lower level of knowledge of these situations (*n* = 97; 36.5%). The differences between the groups were statistically significant (*p* < 0.01) in all items on the instrument.

The results of Student’s *t*-test for independent samples then showed differences between the groups obtained from the cluster analysis, and its value for the sources of information most widely used to gain knowledge about gender-based violence. The group with greater knowledge of situations of gender-based violence or sexual harassment had a higher valuation for sources of information related to the press (t_266_ = −2.697; *p* < 0.01), training received (t_266_ = −2.589; *p* < 0.05), and own experience (t_266_ = −3.858; *p* < 0.01) or that of someone close (t_266_ = −6.064; *p* < 0.01) against the group with less knowledge. For the remaining sources of information, both groups had similar values (see Table 3).

With regard to possible situations of risk of gender-based violence, the students with a greater knowledge of gender-based violence and/or sexual harassment stated that at the boyfriend/girlfriend stage (t_266_ = −3.370; *p* < 0.01), during the breakup of this stage (t_266_ = −2.104; *p* < 0.05), in couples with children (t_266_ = −2.793; *p* < 0.01), or during the process of the divorce/separation of a marriage (t_266_ = −3.454; *p* < 0.01), there is a greater risk of suffering sexual violence and/or sexual harassment, as opposed to those with less knowledge of these situations (see Table 4).

### 4.4. Results for Knowledge of Situations of Violence in the Rural and Urban Environments

The results showed that 68% of the students belonged to groups of a low knowledge in an urban environment compared to 32% that belonged to a group of low knowledge in a rural environment; 71% belonged to groups with a high knowledge in an urban environment compared to 28% who belonged to groups with a high knowledge in a rural environment. However, the association of the variables related to a knowledge of situations of gender-based violence and the place of residence of the participants was not significant (χ^2^(1, 266) = 0.541, *p* = 0.578; Cramer’s V = 0.037; *p* = 0.541).

### 4.5. Results for Knowledge of Situations of Violence, Sources of Information, and Situations of Risk According to Gender

Regarding gender (see Table 5), no differences were detected in the sources of information between men and women. However, some differences were detected in situations of risk (t_267_ = 21,984; *p* < 0.05) and in knowledge of situations of violence (t_268_ = 2.447; *p* < 0.05), both in favour of higher levels on the part of women versus men.

## 5. Discussion

The results obtained by this study provide meaningful data on the beliefs of undergraduate students on gender-based violence and sexual harassment in the university environment. The students taking part have stated that the knowledge they have of gender-based violence comes mainly from sources of information related to communications media such as TV news and the print press, and also from educational activity, such as talks received or study projects related to the subject [12,25,30,44].

On the other hand, the least commonly used sources were those concerned with obtaining knowledge through their own experience or the experience of a friend or family member. With these results to hand, the fact that television and the press are the primary sources of information should not be considered positive, as this type of information, aimed at popular consumption, may not always be appropriate, or completely objective and/or correct [22]. Nonetheless, it can be considered very positive that training given by experts in the subject is the second most common source of knowledge on gender-based violence. This highlights positive results and supports the ideas presented by various authors about the need for education in initial training [39]. However, it is necessary to take into account the existence of great dissimilarities among the different education/training programs in different Spanish universities [36], and it is important to conduct a deep analysis into the diverse programs and their benefits. Some research points to the intervention and training of students as a tool for the prevention of risk situations [41].

The direct experience of these situations, whether by individuals or persons close to them, comes last as a source of knowledge about violence. Of course, although direct experience is among the least common sources, it is a matter of concern that there is evidence that these situations do in fact exist.

With respect to intimate relationships, students say that the situations of greatest risk of acts of gender-based violence or sexual harassment among their circle tend to arise during divorce or during the breakup of a couple, whether they are boyfriend/girlfriend, or a married couple. In fact, the students say that they are very familiar with certain subtler forms of gender-based violence (e.g., situations of jealousy or feelings of possessiveness, control of the partner, sexist remarks, or psychological attack, rather than other, more open or aggressive acts (e.g., preferential treatment or academic favors in return for sexual favors, unwanted kissing and/or touching, physical assaults, or the sending of threatening or intimidating messages or emails). In this sense, other authors indicated that, on occasions, some male students expected some kind of sexual relationship if they provided support or favors to a partner, so these students justify some forms of sexual harassment [10]. These authors add that the participants tried to justify and link cases of gender-based violence within the university, indicating the inappropriate behaviors or clothing of women and the lack of personal prevention measures as possible causes of gender-based violence.

## 6. Conclusions

In conclusion, it was found that the perception of situations of risk in relationships was greater when students had a greater knowledge of gender-based violence or sexual harassment within their own circle [32,33]. However, a higher perception of situations of risk in relationships was not affected by the source of information (TV, radio, etc.) It could be assumed that when a student is familiar with a situation of gender-based violence in their own environment they are more alert to these situations, and so this knowledge might lead them to be more aware of certain types of violence which might go unnoticed by a student whose knowledge of violence comes from indirect sources (e.g., television).

In fact, the study identified two groups with clearly distinguished profiles that depended on their knowledge of situations of gender-based violence. One group with a high level of knowledge about these situations because they have at some time experienced or been aware of situations of gender-based violence among people close to them, and a second group with less knowledge of these violent situations, stemming from a more limited experience of these situations [1,12]. As expected [28], a greater knowledge of gender-based violence or sexual harassment is linked to experience of this kind of situation by the student.

The profile with greater knowledge of gender-based violence and/or sexual harassment state that, at the boyfriend/girlfriend stage [21], during the breakup of such a relationship or a marriage, and when a couple has children, there is a greater risk of suffering gender-based violence and/or sexual harassment, as opposed to the group with less knowledge of gender-based violence [2,45]. Despite this, and regardless of the knowledge profiles on the gender-based violence of students (high vs. low), stages that can also be seen as critical or with a risk of violence are the periods following the breakup of a relationship or a marriage [17], and even situations within the marriage itself [22].

Some authors opt for different measurements that they consider to be useful in the treatment of gender violence in universities. Among them are as follows: (a) integrate specific units on gender-based violence within the university; (b) develop specialized courses to address gender violence in university classrooms; and/or (c) develop programs to qualify students who are interested in acting as educators of their own classmates and advocates of violence and sexual assault. This theory has also been supported by Valls et al. [1], as we have indicated previously.

Finally, the place of residence (rural vs. urban) is not linked to a higher or lower knowledge of situations of gender-based violence. This last result should be treated with caution, since the limitations of the study sample may mean that it fails to identify what is considered a risk factor for suffering violence. In this regard, the literature [26,27] looks at the particular vulnerability of women in the rural environment. All of this shows, as a negative finding, that gender-based violence and/or sexual harassment, whether suffered directly or by persons close to the subject, happens or has happened to students from both rural and urban environments.

With regard to gender, it is evident that university women show a superior perception of situations of risk and knowledge of situations of violence compared to their male peers. This result coincides with the study of Castillo-Acobo and Choqque-Soto [13], in the universities of Arequipa (Peru). Despite the above, in our study the sources of information are similar between men and women.

We feel it is important to underline the need for the analysis we have carried out, as it should provide an awareness that in turn leads to a rethinking of the accepted landscape, in order to reshape existing social constructs with regard to women and men. To this end, we should remember that the educational environment at all levels is the prime ground that must be sown with the new ideas that need to be introduced. However, given the actual state of affairs, it is essential to involve university students in this social transformation, as these current students will one day govern the country, deliver justice, teach, heal, etc., the citizens of the future. Hence the importance of having a gender perspective present at all stages of learning, including as an aim of lifelong learning. No social transformation on this scale is possible without the involvement of those stepping up the generational ladder in all professional, social, and political fields.

### Limitations and Future Lines of Research

Among the main limitations of this study is the fact that the sample size does not allow the results to be generalized to other populations. With regard to the methodology, the cross-curricular nature of the study means that no predictive analysis could be carried out on the variables studied. Likewise, the use of quantitative self-reporting, common in this type of research, could be complemented with qualitative instruments, which would allow for a greater understanding of this type of situation. Despite this, the data and results obtained are valuable and move forward our knowledge in the field of sexual violence and/or harassment.

Future research hopes to increase the sample size and the representation of the reference populations, including participants from other fields of study. Considering the above, the intention is to carry out studies into the extent of this type of situation in the university environment, longitudinal studies to obtain causal results, and the introduction of mixed methodologies (quantitative and qualitative), all aimed at finding out more about violence and/or sexual harassment.

## Figures and Tables

**Table 1:** Reliability and items for the instrument “Situations of Risk in a Relationship” (S-SRR) and “Knowledge of Situations of Gender-based Violence” (S-KSGV).

Instrument	Items	
S-SRR (α = 0.757)	SRR1	Prior to marriage
	SRR2	On trying to break up with the partner
	SRR3	During marriage
	SRR4	On having children
	SRR5	During the process of divorce or separation
	SRR6	After divorce or separation
S-KSGV (α = 0.909)	KSGV1	Constant control (of activities undertaken, of who one is with...)
	KSGV2	Jealousy (possessive feelings)
	KSGV3	Threatening or intimidating phone messages or emails
	KSGV4	Physical attack
	KSGV5	Psychological attack
	KSGV6	Sexist remarks
	KSGV7	Pressure to engage in an emotional and/or sexual relationship
	KSGV8	Kissing and/or touching without consent
	KSGV9	Discomfort or fear due to feeling harassed or intimidated
	KSGV10	Obscene remarks, rumors or attacks on sex life
	KSGV11	Preferential treatment or academic favors in exchange for sexual favors
	KSGV12	Demeaning use of Internet images, even as a “joke”

Note: S-SRR = Scale “Risk Situations in a Relationship”; S-KSGV = Scale “Knowledge of Situations of Gender-based Violence”.

**Table 2:** Bivariate correlations between knowledge of situations of violence, sources of information, and situations of risk in a relationship.

	TV	RD	PR	TC	OE	F/FE	S-SRR	S-KSGV
TV	1							
RD	0.361 **	1						
PR	0.305 **	0.421 **	1					
TC	−0.012	−0.036	0.180 **	1				
OE	−0.162 **	0.141 *	−0.038	0.090	1			
F/FE	−0.091	−0.018	0.089	0.144 *	0.417 **	1		
S-SRR	0.040	−0.104	0.098	0.064	0.006	0.232 **	1	
S-KSGV	−0.051	0.057	0.171 **	0.150 *	0.281 **	0.460 **	0.326 **	1

Note: TV = TV news/Television; RD = Radio; PR = Press; TC = Training or chats; OE = Own experience; F/FE = Friends/Family experience; S-SRR = Scale “Risk Situations in a Relationship”; S-KSGV = Scale “Knowledge of Situations of Gender-based Violence”; * = *p* < 0.05; ** = *p* < 0.01.

**Table 3:** Significant differences in the sources of information between the groups with a higher and lower knowledge of situations of violence.

Sources of Information	Profile	*n*	M	SD	*t*	*p*	*d*
TV	Lower	97	3.40	1.067	−0.362	0.717	0.05
	Higher	169	3.45	1.011			
RD	Lower	97	2.13	1.187	−1.187	0.236	0.16
	Higher	169	2.30	1.062			
PR	Lower	97	2.29	1.163	−2.697	0.007 **	0.34
	Higher	169	2.67	1.100			
TC	Lower	97	3.02	1.233	−2.589	0.010 *	0.33
	Higher	169	3.41	1.141			
OE	Lower	97	2.10	1.271	−3.858	0.000 **	0.50
	Higher	169	2.79	1.456			
F/FE	Lower	97	1.77	1.221	−6.064	0.000 **	0.75
	Higher	169	2.77	1.402			

Note: TV = TV news/Television; RD = Radio; PR = Press; TC = Training or chats; OE= Own experience; F/FE = Friends/Family experience; *n* = sample; M = mean; SD = standard deviation; * *p* < 0.05; ** *p* < 0.01; *d* = Cohen’s *d*.

**Table 4:** Significant differences in situation of risk in a relationship between groups with a higher and lower knowledge of situations of violence.

Situation of Risk	Profile	*n*	M	SD	*t*	*p*	*d*
Boyfriend/Girlfriend stage	Lower	97	3.19	0.950	−3.370	0.001 **	0.43
	Higher	169	3.60	0.966			
When wishing to break up	Lower	97	4.20	0.886	−2.104	0.036 *	0.27
	Higher	169	4.43	0.843			
Within marriage	Lower	97	3.42	1.329	−1.575	0.116	0.21
	Higher	169	3.69	1.306			
With children	Lower	97	3.47	0.936	−2.793	0.006 **	0.36
	Higher	169	3.81	0.951			
During divorce/separation	Lower	97	3.81	1.034	−3.454	0.001 **	0.45
	Higher	169	4.24	0.915			
After divorce/separation	Lower	97	3.91	1.091	−1.271	0.205	0.16
	Higher	169	4.08	1.024			

Note: *n* = sample; M = mean; SD = standard deviation; * = *p* < 0.05; ** = *p* < 0.01; *d* = Cohen’s *d*.

**Table 5:** Significant differences in knowledge of situations of violence, sources of information and other situations according to gender.

Measure	Gender	*n*	M	SD	*t*	*p*	*d*
TV	Woman	178	3.40	1.049	−0.463	0.644	0.07
	Man	90	3.47	1.019			
RD	Woman	178	2.26	1.110	0.560	0.576	0.07
	Man	90	2.18	1.118			
PR	Woman	178	2.53	1.131	0.040	0.968	0.07
	Man	90	2.52	1.154			
TC	Woman	178	3.28	1.159	0.380	0.704	0.05
	Man	90	3.22	1.261			
OE	Woman	178	2.59	1.420	1.028	0.305	0.13
	Man	90	2.40	1.444			
F/FE	Woman	178	2.40	1.363	0.082	0.935	0.01
	Man	90	2.39	1.527			
S-SRR	Woman	178	3.94	0.619	2.447	0.016 *	0.34
	Man	90	3.71	0.804			
S-KSGV	Woman	177	2.87	0.951	1.984	0.048 *	0.26
	Man	90	2.62	0.978			

Note: TV = TV news/Television; RD = Radio; PR = Press; TC = Training or chats; OE = Own experience; F/FE = Friends/Family experience; S-SRR = Scale “Risk Situations in a Relationship”; S-KSGV = Scale “Knowledge of Situations of Gender-based Violence”; *n* = sample; M = mean; SD = standard deviation; * = *p* < 0.05; *d* = Cohen’s *d.*
